# Assessment of Influenza Severity in Bhutan by Using WHO Framework Pandemic Influenza Severity Assessment (PISA): An Implementation Research Study

**DOI:** 10.1111/irv.70095

**Published:** 2025-03-20

**Authors:** Tshering Dorji, Kunzang Dorji, Vishal Chettri, Sonam Gyeltshen, Holly Sadler

**Affiliations:** ^1^ National Influenza Centre (NIC) Royal Centre for Disease Control Thimphu Bhutan; ^2^ Food and Nutrition Laboratory (FNL) Royal Centre for Disease Control Thimphu Bhutan; ^3^ Consultant to the World Health Organization Geneva Switzerland

**Keywords:** Bhutan, influenza, pandemic preparedness, PISA framework, surveillance, WHO average curve method

## Abstract

**Background:**

Influenza presents a significant global health challenge, with seasonal epidemics causing 3 to 5 million cases of severe illness and 290,000 to 650,000 respiratory deaths annually. In Bhutan, the highest rates of influenza‐associated hospitalizations were observed among children under 5 years of age emphasizing the need for robust surveillance and preparedness.

**Objective:**

This study aims to assess influenza severity in Bhutan using the World Health Organization's (WHO) Pandemic Influenza Severity Assessment (PISA) framework. By integrating syndromic and influenza‐specific data, we establish national‐level baseline and threshold values for influenza activity.

**Methods:**

The WHO Average Curve Method was employed to establish seasonal and intensity thresholds, categorizing influenza severity based on historical data from 2016 to 2019 and 2023.

**Results:**

Analysis of influenza activity revealed near‐continuous activity with two annual peaks. Thresholds for epidemic, moderate, high, and extraordinary levels of transmissibility and morbidity were determined. The 2019 season exhibited the highest transmissibility and morbidity, with significant variability in intensity across different seasons.

**Conclusion:**

The study demonstrates the effectiveness of the PISA framework in assessing influenza severity in Bhutan. The established thresholds provide a valuable tool for public health decision‐making, enhancing the country's preparedness for both seasonal and pandemic influenza. These findings underscore the importance of maintaining and adapting surveillance systems to monitor influenza activity year‐round.

## 1 Introduction

Seasonal influenza poses a significant global health challenge, causing considerable morbidity and mortality. The seasonal influenza epidemics result in 3 to 5 million cases of severe illness, and 290,000 to 650,000 respiratory deaths annually [1]. In Bhutan, influenza‐associated respiratory hospitalizations were estimated at 50 per 100,000 persons (95% CI: 45–55) in 2015 and 118 per 100,000 persons (95% CI: 110–127) in 2016, with the highest rates observed among children under 5 years of age [2].

Historically, influenza has caused four global pandemics in 1918 (H1N1), 1957 (H2N2), 1968 (H3N2), and 2009 (H1N1) [3]. The virus's constant genetic changes pose a continual threat of novel strains. The 2009 H1N1 pandemic revealed significant gaps in preparedness, prompting advancements in surveillance, focused research, and risk assessment tools [3]. Consequently, the 2011 World Health Assembly recommended developing severity assessment measures for influenza epidemics, leading to the adoption of the Pandemic Influenza Severity Assessment (PISA) framework. This framework evaluates influenza severity by integrating measures of transmissibility, disease seriousness, and healthcare impact, based on historical data, facilitating comparisons during both epidemic and pandemic periods [4].

The COVID‐19 pandemic in 2020 underscored the necessity of enhancing the World Health Organization's (WHO) severity assessments for non‐influenza viruses and differentiating between syndromic and pathogen‐specific assessments. This led to the expansion of the PISA framework to include non‐influenza respiratory viruses. A Technical Working Group (TWG) reviewed PISA's performance during COVID‐19, exploring parameter choices and threshold‐setting differences between influenza and SARS‐CoV‐2. In 2024, the revised PISA framework was published, allowing for continuous assessment of influenza and syndromic respiratory illness activity relative to historical data [5]. In addition, the impact indicator was split into two separate but related indicators; one which measures the amount of morbidity and mortality caused by the epidemic or pandemic, and another which measures the impact of this demand on healthcare capacity and function.

Bhutan, a small landlocked country in South Asia with an estimated population of approximately 770,276, operates an integrated three‐tier healthcare system consisting of a National Referral Hospital (NRH) at its apex, regional and district hospitals, and community‐level facilities (Primary Health Centres and Thromde Health Centres) [6, 7]. The country is administratively divided into 20 districts, with referral hospitals strategically located in the western, central, and eastern regions to facilitate equitable healthcare access. Influenza surveillance in Bhutan is conducted through two complementary national systems. The National Early Warning, Alert, and Response Surveillance (NEWARS) system provides syndromic surveillance data, including acute respiratory infections (ARI), from all health centers nationwide. The COVID‐19 Integrated Influenza Surveillance system is a sentinel‐based system that monitors Influenza‐Like Illness (ILI) and Severe Acute Respiratory Infection (SARI) at designated hospitals, incorporating virological testing to confirm influenza cases. These surveillance systems serve as the primary data sources for assessing influenza epidemiology, including transmissibility, morbidity, and mortality.

In this study, we use the PISA framework to describe the epidemiological situation of influenza in Bhutan using both syndromic and influenza‐specific historical data. Establishing national‐level baseline and threshold values for influenza helps determine whether the current season differs in timing and severity from historical data. This approach significantly contributes to Bhutan's pandemic preparedness plan, offering valuable insights to strengthen influenza surveillance and response.

## 2 Methods

### 2.1 Influenza Surveillance System in Bhutan

Prior to 2008, Bhutan lacked a systematic surveillance mechanism for detecting pathogens associated with ARIs, despite the substantial morbidity attributed to these infections. In 2008, the Royal Centre for Disease Control (RCDC), formerly known as the Public Health Laboratory, collaborated with the Department of Virology at the Armed Forces Research Institute of Medical Sciences (AFRIMS) in Bangkok, Thailand, to initiate Bhutan's first influenza virological surveillance program. Initially, this program operated at three locations: Jigme Dorji Wangchuk National Referral Hospital in Thimphu, Paro Hospital, and Punakha Hospital [8].

Following the 2009 H1N1 pandemic, the surveillance network expanded to nine sites with the addition of hospitals in Phuentsholing, Trongsa, Tsirang, Gelephu, Mongar, and Trashigang. By 2010, two additional sites (Samtse and Samdrup Jongkhar hospitals) were incorporated, thereby establishing an eleven‐site sentinel network to monitor ILI in Outpatient Departments (OPDs) and SARI in Inpatient Departments (IPDs) [8]. Concurrently, Bhutan implemented the Notifiable Disease Surveillance System (NDSS) in 2010, which was later upgraded to the NEWARS system. NEWARS is a web‐based platform supporting nationwide syndromic surveillance, requiring weekly reporting of ARI cases from all health centers. It also incorporates indicator‐based surveillance for 26 additional notifiable diseases and ad hoc event‐based surveillance [9].

**FIGURE 1:**
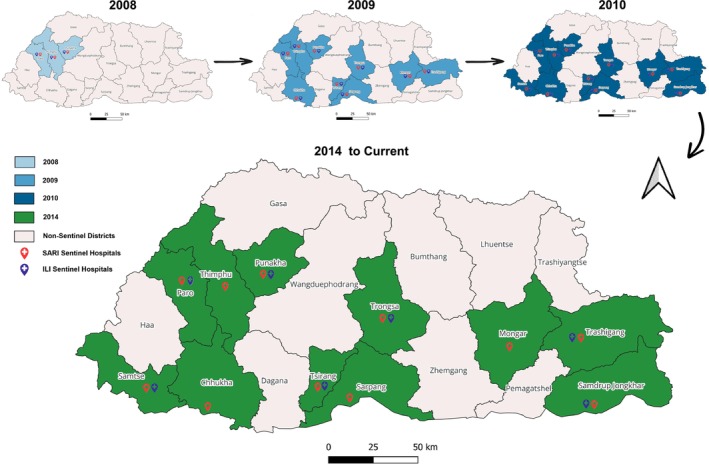
Influenza sentinel site progression in Bhutan.

In 2014, national guidelines for sentinel influenza surveillance were revised, streamlining ILI surveillance to seven hospitals, while SARI surveillance continued across all 11 sites (Figure 1) [10]. During the COVID‐19 pandemic, the existing influenza surveillance infrastructure was leveraged to incorporate SARS‐CoV‐2 detection, allowing for efficient integration of pandemic response efforts. In 2022, the system was further revised as the COVID‐19 Integrated Influenza Surveillance system, incorporating emerging SARS‐CoV‐2 epidemiological trends and optimizing laboratory workflows for concurrent influenza and SARS‐CoV‐2 testing [11].

### 2.2 Data Sources

Two national surveillance systems, NEWARS and the COVID‐19 integrated influenza surveillance system, were used to assess influenza severity indicators. The NEWARS system collects weekly data on notifiable diseases and syndromes, including ARI. An ARI case is defined as an individual presenting with a measured or reported fever of ≥38°C, along with a cough or sore throat, with symptom onset within the past 7 days. Data from all health centers are aggregated weekly, providing population‐level trends in respiratory morbidity.

The COVID‐19 integrated influenza surveillance system uses a sentinel‐based approach to monitor ILI and SARI across designated hospitals. The ILI case definition follows the updated WHO standard case definition, categorizing a case as an individual with an acute respiratory infection, a fever of ≥38°C, and a cough, with symptom onset within the past 10 days. At each of the seven designated sentinel sites (Figure 1), focal points collect and report weekly data on the total number of ILI consultations as well as the total number of outpatient visits. Each sentinel site also collects 10 to 15 respiratory specimens per week for virological analysis.

The SARI surveillance component operates across 11 geographically distributed sentinel sites (Figure 1). Patients meeting the SARI case definition undergo swabbing for testing influenza and other respiratory viruses unless they decline sample collection. A SARI case is defined as an individual with an acute respiratory infection, a history of fever (or a measured temperature of ≥ 38°C), a cough, symptom onset within the previous 10 days, and a requirement for hospitalization. Weekly reports include data on SARI admissions along with the total number of inpatient admissions.

### 2.3 Parameter Selection

To assess transmissibility indicator, ARI data from NEWARS and ILI data from the COVID‐19 integrated influenza surveillance system were used. The weekly number of ARI cases reported in NEWARS was included as a transmissibility parameter due to its nationwide coverage, capturing syndromic respiratory illness trends across all health centers. However, since ARI is not specific to influenza, additional parameters were incorporated to improve specificity. To complement transmissibility assessments, the ILI positivity rate and an ILI composite parameter were derived from ILI data collected at sentinel sites. The ILI composite parameter was calculated as the product of the weekly proportion of ILI cases per 100 outpatient visits and the ILI positivity rate, integrating both syndromic surveillance and laboratory‐confirmed data. This combined approach enhances the specificity of transmissibility estimates by leveraging the broad surveillance coverage of ARI data while refining estimates with virologically confirmed influenza trends. Morbidity and mortality assessments were based on SARI data from the 11 sentinel sites, using the SARI positivity rate and the SARI composite parameter. A summary of the parameters and their calculation methods is provided in Table 1.

**TABLE 1:** Indicators and parameters used in the PISA in Bhutan with source of data and method of calculation to establish thresholds.

Indicator	WHO recommended parameters	Parameters considered for Bhutan	Data source	Calculation
Transmissibility	Weekly ARI cases as a proportion of total visits or incidence rates	No. of weekly ARI cases reported	NEWARS (Web‐based, Nation‐wide)	No. of ARI cases reported in a week
	Weekly ILI or ARI cases as a proportion of total visits or incidence rates	Weekly ILI cases as a proportion of total visits (ILI proportion)^a^	COVID‐19 integrated influenza surveillance (ILI, 7 sites)	No. of weekly ILI cases reported per 100 patients visiting OPD
	Percentage positivity from specific syndromic presentations (e.g., ILI, ARI, MAARI)	Weekly influenza percentage positivity from ILI (ILI positivity)	COVID‐19 integrated influenza surveillance (ILI, 7 sites)	$\frac{\mathrm{ILI}\;\text{positive for}\;\mathrm{Flu}}{\text{Total}\;\mathrm{ILI}\;\text{sample tested}}\times 100$
	Composite (product) of weekly ILI proportions or rates and weekly percentage positivity for influenza	Composite (product) of weekly ILI proportions and weekly positivity rate for influenza	COVID‐19 integrated influenza surveillance (ILI, 7 sites)	ILIProportion×ILIPositivity percent
Morbidity and mortality	SARI proportion or influenza‐confirmed SARI proportion of all hospital or ICU admissions.	Weekly SARI cases as a proportion of total hospital admissions (SARI proportion)^a^	COVID‐19 integrated influenza surveillance (SARI 11 sites)	No. of weekly SARI cases reported per 100 hospitalizations
	SARI proportion or influenza‐confirmed SARI proportion of all hospital or ICU admissions	Influenza‐confirmed SARI (SARI positivity percent)	COVID‐19 integrated influenza surveillance (SARI, 11 sites)	$\frac{\text{SARI positive}\;}{\text{Total SARI tested}}\times 100$
	Composite (product) of weekly SARI rate and weekly percentage positivity rates of SARI cases for influenza	Composite (product) of weekly SARI rate and weekly positivity rates of SARI cases for influenza	COVID‐19 integrated influenza surveillance (SARI, 11 sites)	SARI Proportion×SARI Positivity percent

^a^ Used in the calculation of the composite parameter and not as an independent indicator.

All extracted data were assessed for completeness. Consistent and complete reporting was only available from 2016 onward, and therefore, historical datasets were curated for a five‐year period, covering data from 2016 to 2019, and 2023. Incomplete datasets prior to 2016 and those from 2020 to 2022 were excluded from the analysis due to inconsistent datasets and disruptions in routine influenza surveillance during the COVID‐19 pandemic. For the current influenza season (2024), all data available at the time of this study (up to epidemiological Week 28) were utilized to evaluate influenza activity.

### 2.4 Statistical Analysis

To set thresholds for transmissibility and morbidity and mortality, the recommended methods include the WHO Average Curve Method (ACM) [12] and the Moving Epidemic Method (MEM) [12] which both set fixed thresholds around peak epidemic values, with the MEM being more sensitive to the number of historical years used in the calculations. Additionally, the percentile method and country‐specific statistical approaches may be employed based on the specific attributes of the surveillance data [4].

In our analysis, we chose the WHO ACM to establish seasonal and alert thresholds for the evaluated parameters. We adopted a two‐wave model with a single set of thresholds to accommodate the potential bimodal distribution of influenza activity observed during a single season. Under this model, historical data spanning the entire calendar year are used to construct an average epidemic curve considering both the primary and secondary peaks. The thresholds designed to categorize intensity levels ranging from “no activity or below epidemic threshold” to “low”, “moderate”, “high” and “extraordinary” were calculated following the guidelines outlined in the second edition of the PISA framework using WHO web application tool (https://worldhealthorg.shinyapps.io/averagecurves/) [5]. The epidemic threshold was determined by calculating the historical median for all weeks, smoothed over a three‐week period. The geometric mean was then applied to refine the curve. The 40%, 90%, and 97.5% confidence intervals of a normal distribution around the peak of the average curve were then used to define moderate, high, and extraordinary thresholds as per the PISA guideline [5].

## 3 Results

### 3.1 Comparison of Parameters to Monitor Influenza Activities

**FIGURE 2:**
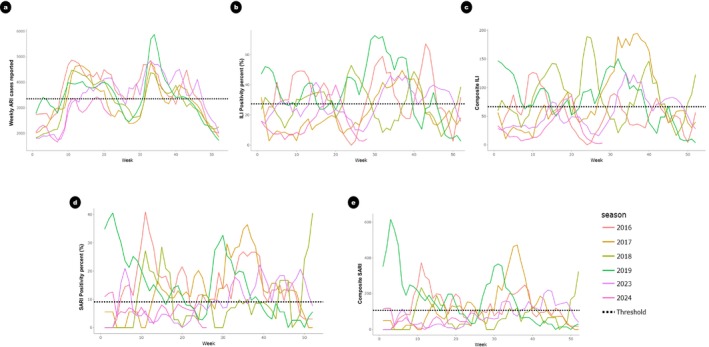
Alignment of yearly epidemiological curves from Weeks 1–52 along with the seasonal threshold set for each parameter. a) Weekly ARI cases reported, b) ILI Positivity percent (%), c) Composite ILI, d) SARI Positivity percent (%), and e) Composite SARI.

The epidemiological curves of the 2016, 2017, 2018, 2019, 2023, and 2024 (up to Week 28) influenza seasons were aligned based on the epidemiological weeks to monitor influenza activity and seasonality (Figure 2). While ARI cases displayed a clear bimodal seasonality, with peaks typically occurring between February to March and August to September, influenza‐specific datasets from SARI and ILI sentinel surveillance, such as the proportion of cases, percent positivity, and composite parameters, showed influenza activity with peaks that fluctuated in timing each year, making it challenging to define a consistent seasonal period.

Despite this variation, our data suggested the presence of two peaks per year on average. To account for this variability, we adopted a two‐wave model with a single set of thresholds for the entire year, allowing for a consistent method of comparing influenza activity across seasons. The thresholds were based on the highest observed peak each season, as this approach provides a more conservative estimate of influenza activity that is less influenced by fluctuations in the secondary peaks.

### 3.2 Establishment of Thresholds

The different thresholds and intensity levels were determined by WHO ACM using the two‐wave model with a single set of thresholds established for the entire year. Four threshold levels were established (Table 2), defining five intensity levels as described above.

**TABLE 2:** Threshold level for influenza severity.

Threshold level	Transmissibility			Morbidity and mortality	
	ARI cases^a^	ILI Positivity (%)^b^	Composite ILI	SARI Positivity (%)^c^	Composite SARI
Epidemic	3340	26.4	58.3	9.1	107.0
Moderate	4620	53.8	128.0	23.0	228.0
High	5560	72.4	198.0	44.7	505.0
Extraordinary	6040	81.8	240.0	65.1	695.0

^a^ No. of weekly ARI cases reported to National Early Warning, Alert, and Response Surveillance (NEWARS) system reported by all health centers in Bhutan.

^b^ Influenza positivity percent (%) from total weekly ILI samples tested.

^c^ Influenza positivity percent (%) from total weekly SARI samples tested.

### 3.3 Transmissibility

**FIGURE 3:**
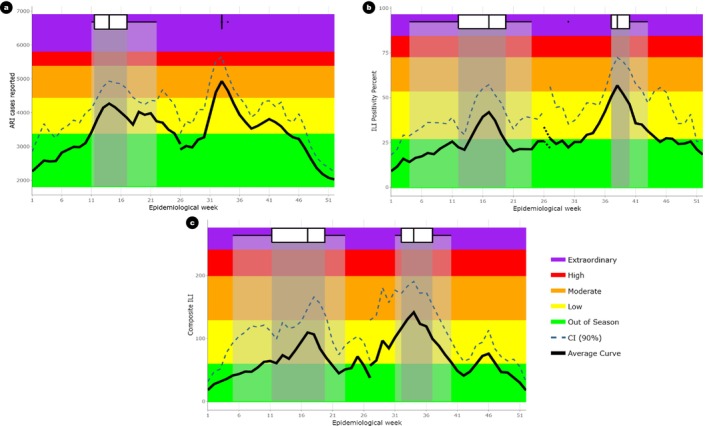
Influenza transmissibility in Bhutan by epidemiological week. a) ARI cases reported, b) ILI positivity percent (%), and c) Composite ILI. Box plots represent the distribution across the peak weeks. The center line indicates the median peak week, the box shows the interquartile range, and the lines extend to represent the full range of previous peak weeks.

Three data sets from two surveillance systems were used to assess the transmission of influenza in Bhutan. The total ARI cases reported by all health centers in Bhutan, the proportion of ILI cases per 100 patients visiting 7 ILI sentinel hospitals and the composite ILI parameter were used to assess transmissibility. Using ARI cases data from NEWARS system, the thresholds for epidemic, moderate, high and extraordinary disease transmission were set at 3340, 4620, 5560, and 6040 ARI cases reported by health centers throughout the country respectively. Surveillance data from the COVID‐19 Integrated Influenza Surveillance provided a composite measure of transmissibility: (number of people with ILI per 100 OPD consultations) × (Influenza positivity percent). The thresholds for epidemic, moderate, high and extraordinary transmission for ILI composite parameters were calculated to be 58.3, 128.0, 198.0 and 240.0 respectively. The detailed thresholds are described in Table 2 and the influenza transmissibility activity by epidemiological week is shown in Figure 3.

### 3.4 Morbidity and Mortality

**FIGURE 4:**
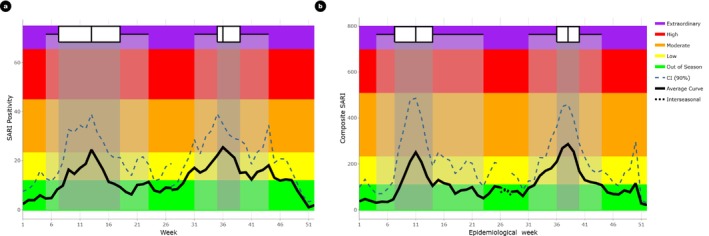
Influenza morbidity and mortality in Bhutan by epidemiological week. a) SARI positivity percent (%) and b) Composite SARI. Box plots represent the distribution across the peak weeks. The center line indicates the median peak week, the box shows the interquartile range, and the lines extend to represent the full range of previous peak weeks.

To assess level of severe disease, SARI sentinel surveillance, a part of COVID‐19 integrated influenza surveillance system was used. Two parameters, SARI percent positivity for influenza ($\frac{\mathrm{No}\;\text{of SARI samples testing positive for influenza}}{\text{Total SARI samples tested}}\times 100$) and the composite SARI (SARI proportion of total inpatients × SARI positivity rate) was used. Using the SARI percent positive dataset, the thresholds for epidemic, moderate, high, and extraordinary were set at 9.1, 23.0, 44.7, and 65.1 (Table 3). For the composite SARI, the thresholds for epidemic, moderate, high, and extraordinary were set at 107.0, 228.0, 505.0, and 695.0 respectively. The thresholds for influenza morbidity and mortality described using SARI proportion and composite SARI are shown in Table 2 and Figure 4.

**TABLE 3:** Influenza activity and intensity levels during the different seasons.

Parameter		Wave characteristics	2016		2017		2018		2019		2023		2024
			1	2	1	2	1	2	1	2	1	2	1
Transmissibility	ARI cases	Peak week	11	43	13	34	11	34	14	34	20	35	18
		Peak value	4870	4480	4660	4590	4470	4330	4030	5870	4130	4700	3420
		Wave intensity	**M**	**L**	**M**	**L**	**L**	**L**	**L**	**H**	**L**	**M**	**L**
ILI Positivity		Peak week	11	43	19	37	23	40	23	30	15	38	20
		Peak value	49.3	67.2	19.7	49.6	46.5	48.9	45.1	72.7	41.6	46.1	39.9
		Wave intensity	**L**	**M**	**BB**	**L**	**L**	**L**	**L**	**H**	**L**	**L**	**L**
Composite ILI		Peak week	10	31	19	37	23	40	5	32	15	34	20
		Peak value	127.0	89.5	92.6	195.0	154.0	147.0	123.0	151.0	75.1	126	85.7
		Wave intensity	**L**	**L**	**L**	**M**	**M**	**M**	**L**	**M**	**L**	**L**	**L**
Morbidity and mortality	SARI Positivity	Peak week	11	35	23	36	15	44	5	31	6	39	19
		Peak value	41.0	26.8	18.1	36.4	28.5	16.7	30.1	25.5	21.0	22.2	8.3
		Wave intensity	**M**	**M**	**L**	**M**	**M**	**L**	**M**	**M**	**L**	**L**	**BB**
Composite SARI		Peak week	11	38	23	36	11	40	4	31	6	44	15
		Peak value	373.0	249.0	154.0	472.0	234.0	125.0	556.0	366.0	114.0	219.0	71.4
		Wave intensity	**M**	**M**	**L**	**M**	**M**	**L**	**H**	**M**	**L**	**L**	**BB**

BB, Below Baseline; L, Low; M, Moderate; H, High; and E, Extraordinary.

*Note:* Intensity classifications were derived by comparing the data from each year with the thresholds outlined in Table 2.

### 3.5 Analysis of the Influenza Activity for the Past Seasons

Across five historical influenza seasons (2016, 2017, 2018, 2019, and 2023) and the current season (2024, up to Week 28), a total of 11 distinct waves were identified through analysis of syndromic, virological, and composite parameters. The wave intensity assessment produced 55 intensity ratings based on three transmissibility parameters and two morbidity and mortality parameters. The ARI surveillance data from NEWARS indicated that 2019 exhibited the highest influenza transmissibility activity among the years studied, with the intensity of the second wave classified as “high.” This was confirmed by the ILI positivity percentage parameter, which also categorized the second wave of the 2019 influenza season as having “high” intensity. However, when assessed using the composite ILI parameter, the 2019 season was classified as a “moderate” intensity season. In terms of morbidity and mortality of the disease, the 2019 season recorded the highest levels, with the first wave classified as “high” intensity and the second wave as only a “moderate” intensity according to the SARI composite parameter. Detailed influenza activity and intensity levels for the different seasons are presented in Table 3.

## 4 Discussion

WHO initiated the PISA framework in March 2017, urging member states to establish influenza baselines and intensity thresholds using surveillance data to monitor and describe the severity of each influenza season using these thresholds [4]. Aligning with this global initiative, our study sought to establish influenza severity assessment thresholds tailored to Bhutan by using data from the NEWARS system and the influenza‐specific COVID‐19 integrated surveillance system. The data sources used in this study have been formally evaluated to have high confidence in terms of timeliness, reliability and data quality through weekly reports published by RCDC (www.rcdc.gov.bt). We identified four key thresholds to signal the onset of the influenza season and differentiate between low, moderate, high, and extraordinary levels of influenza activity, considering both transmission dynamics and disease severity. The choice to use ARI case numbers as one of the measures of influenza transmissibility was based on the strengths of the surveillance system's reporting and coverage. ARI syndromic surveillance in Bhutan offers broader representativeness, as all health centers report weekly ARI cases under NEWARS guidelines. However, its lower specificity can lead to false alerts, as noted in previous studies [12, 13].

Our analysis of weekly data from the COVID‐19 integrated influenza surveillance system revealed that influenza in Bhutan does not follow a distinct seasonal pattern. Instead, it exhibits a bimodal seasonality, with peaks fluctuating in timing each year, making it challenging to define a consistent seasonal period. This contrasts with the typical synchronized influenza epidemics observed during winter in temperate regions of the Northern and Southern Hemispheres [14]. However, this bimodal pattern aligns with previous studies in Bhutan, such as the work by Thapa et al., who documented two annual influenza peaks, with the secondary peak typically occurring between July and September [2]. Other regional studies have also confirmed this unique semi‐annual activity, with distinct peaks in both winter and summer [15, 16, 17]. This pattern is further supported by our laboratory's earlier report showing increased genetic diversity among influenza A(H3N2) strains during peak periods, reinforcing the presence of two distinct annual surges [18]. Given Bhutan's already robust continuous sentinel COVID‐19 surveillance and notifiable disease surveillance systems, this pattern further underscores the importance of maintaining and adapting these systems to effectively monitor and respond to influenza year‐round. The lack of clear seasonality challenges traditional influenza preparedness, requiring flexible vaccination campaigns and public health interventions, potentially targeting the identified peak periods to maximize effectiveness.

The WHO PISA guideline has proven to be a valuable resource and adaptable for integrating and analyzing diverse data sources, facilitating the establishment of standardized thresholds to evaluate influenza severity in Bhutan. Similar experiences were also noted in the establishment of PISA thresholds in countries like Australia [19], Morocco [20], Singapore [21], Democratic Republic of Congo [22] and Mauritius [12]. Our analysis demonstrated strong concordance across various data sources in measuring indicator activity levels throughout the historical seasons studied. Particularly during the 2019 influenza season, the transmissibility indicators consistently reflected similar intensity levels at the seasonal peak, highlighting the practical public health relevance of our findings. The classification of wave intensity into categories such as low, moderate, high, and extraordinary plays a crucial role in guiding public health responses. For instance, during high or extraordinary waves, it becomes imperative to escalate preparedness, including increasing hospital capacity, ensuring the availability of antiviral medications and enhancing public health advocacy programs. By establishing clear parameters, thresholds, and categorizations, we can effectively trigger specific public health actions, such as risk communication, when these thresholds are exceeded.

In conclusion, our study demonstrates that the threshold methodology by ACM, as outlined in the WHO manual, is both easily adoptable and effective for assessing influenza severity. This approach is not only applicable to seasonal influenza but also enhances preparedness for potential pandemic influenza, contributing significantly to the country's pandemic readiness. These findings hold important implications particularly for countries with limited resources, where adapting surveillance and response strategies is crucial for effective public health management.

### 4.1 Limitations

One of the limitations of this study is the inability to establish a threshold for assessing the impact on healthcare capacity due to the lack of data on healthcare usage and workforce absenteeism. The data on ILI and SARI were derived from sentinel surveillance, which may not accurately represent the entire population. In addition, the proportions of consultations for ARI from the NEWARS could not be calculated because of the absence of data on the total number of patients visiting hospitals in Bhutan. However, despite this limitation, tracking ARI attendances remains crucial as it provides potential insights into changes in the clinical presentation of influenza cases.

## Author Contributions

**Tshering Dorji:** conceptualization, investigation, writing – original draft, methodology, validation, visualization, writing – review and editing, software, formal analysis, data curation, investigation, methodology, validation, writing – review and editing, data curation. **Kunzang Dorji:** conceptualization, investigation, writing – original draft, methodology, validation, visualization, writing – review and editing, software, formal analysis, data curation, investigation, methodology, validation, writing – review and editing, data curation. **Vishal Chettri:** investigation, methodology, validation, writing – review and editing. **Sonam Gyeltshen:** investigation, methodology, validation, writing – review and editing, data curation. **Holly Sadler:** investigation, methodology, validation, visualization, writing – review and editing, software, formal analysis, data curation.

## Ethical Approval

The syndromic ARI datasets used in this study were obtained from the NEWARS system, a public health activity coordinated by the RCDC under the Ministry of Health, Bhutan. Influenza‐specific data were obtained from COVID‐19 integrated influenza surveillance as part of the national influenza control program. The study protocol was reviewed by the Research Ethics Board of Health (REBH) in Bhutan **(Ref. No. 2024.43.NW)** and was exempted for REBH ethical approval. This study exclusively utilizes de‐identified aggregate and cumulative data, ensuring that no confidential information potentially identifying individuals are included.

## Conflicts of Interest

The authors declare no conflicts of interest.

## 5 Data Availability Statement

Data are already available in the manuscript in the form of quotations. Raw interview transcripts will not be made available.

### Peer Review

The peer review history for this article is available at https://www.webofscience.com/api/gateway/wos/peer‐review/10.1111/irv.70095.

## References

[1] A. D. Iuliano , K. M. Roguski , H. H. Chang , et al., “Estimates of Global Seasonal Influenza‐Associated Respiratory Mortality: A Modelling Study,” Lancet 391, no. 10127 (2018): 1285–1300. 29248255 10.1016/S0140-6736(17)33293-2 PMC5935243

[2] B. Thapa , K. Roguski , E. Azziz‐Baumgartner , et al., “The Burden of Influenza‐Associated Respiratory Hospitalizations in Bhutan, 2015–2016,” Influenza and Other Respiratory Viruses 13, no. 1 (2019): 28–35, 10.1111/irv.12605. 30137672 PMC6304319

[3] W. N. Harrington , C. M. Kackos , and R. J. Webby , “The Evolution and Future of Influenza Pandemic Preparedness,” Experimental & Molecular Medicine 53, no. 5 (2021): 737–749. 33953324 10.1038/s12276-021-00603-0 PMC8099712

[4] Pandemic Influenza Severity Assessment (PISA): A WHO Guide to Assess the Severity of Influenza Epidemics and Pandemics (World Health Organization, 2017).

[5] Pandemic Influenza Severity Assessment (PISA): A WHO Guide to Assess the Severity of Influenza in Seasonal Epidemics and Pandemics, Second Edition. Geneva: World Health Organization, Second ed. (World Health Organization, 2024. Licence: CC BY‐NC‐SA 3.0 IGO).

[6] Statistical Yearbook of Bhutan , “National Statistics Bureau, Royal Government of Bhutan,” (2023), https://www.nsb.gov.bt/publications/statistical‐yearbook/.

[7] Annual Health Bulletin ,.Policy and Planning Division, Ministry of Health, Thimphu, Bhutan,” (2024).

[8] S. Wangchuk , B. Thapa , S. Zangmo , R. G. Jarman , P. Bhoomiboonchoo , and R. V. Gibbons , “Influenza Surveillance From November 2008 to 2011; Including Pandemic Influenza A(H1N1)pdm09 in Bhutan,” Influenza and Other Respiratory Viruses 7, no. 3 (2013): 426–430. 22813389 10.1111/j.1750-2659.2012.00409.x PMC5779828

[9] National Early Warning , Alert, and Response Surveillance Guideline, Second ed. (Royal Centre for Disease Control, Ministry of Health, Bhutan, 2018).

[10] Operational Guideline for Influenza‐Like Illness and Severe Acute Respiratory Infection, Royal Centre for Disease Control, Ministry of Health, Bhutan, Second ed. (Royal Centre for Disease Control, Ministry of Health, 2014).

[11] “COVID‐19 Integrated Influenza Surveillance Guideline,” Second edition, Royal Centre for Disease Control, 2022, ISBN 978–99980–45‐07‐1.

[12] M. Teeluck and A. Samura , “Assessing the Appropriateness of the Moving Epidemic Method and WHO Average Curve Method for the Syndromic Surveillance of Acute Respiratory Infection in Mauritius,” PLoS ONE 16, no. 6 (2021): e0252703. 34081752 10.1371/journal.pone.0252703 PMC8174728

[13] J. S. Casalegno , D. Eibach , M. Valette , et al., “Performance of Influenza Case Definitions for Influenza Community Surveillance: Based on the French Influenza Surveillance Network GROG, 2009–2014,” Eurosurveillance 22, no. 14 (2017): 30504. 28422004 10.2807/1560-7917.ES.2017.22.14.30504 PMC5388124

[14] B. S. Finkelman , C. Viboud , K. Koelle , M. J. Ferrari , N. Bharti , and B. T. Grenfell , “Global Patterns in Seasonal Activity of Influenza A/H3N2, A/H1N1, and B From 1997 to 2005: Viral Coexistence and Latitudinal Gradients,” PLoS ONE 2, no. 12 (2007): e1296. 18074020 10.1371/journal.pone.0001296 PMC2117904

[15] K. Bloom‐Feshbach , W. J. Alonso , V. Charu , et al., “Latitudinal Variations in Seasonal Activity of Influenza and Respiratory Syncytial Virus (RSV): A Global Comparative Review,” PLoS ONE 8 (2013): e54445. 23457451 10.1371/journal.pone.0054445 PMC3573019

[16] S. Hirve , L. P. Newman , J. Paget , et al., “Influenza Seasonality in the Tropics and Subtropics – When to Vaccinate?,” PLoS ONE 11, no. 4 (2016): e0153003. 27119988 10.1371/journal.pone.0153003 PMC4847850

[17] S. Caini , W. Andrade , S. Badur , et al., “Temporal Patterns of Influenza A and B in Tropical and Temperate Countries: What Are the Lessons for Influenza Vaccination?,” PLoS ONE 11, no. 3 (2016): e0152310. 27031105 10.1371/journal.pone.0152310 PMC4816507

[18] T. Dorji , K. Dorji , and S. Gyeltshen , “Evolution of Influenza A(H3N2) Viruses in Bhutan for Two Consecutive Years, 2022 and 2023,” Influenza and Other Respiratory Viruses 18, no. 10 (2024): e70028. 39443295 10.1111/irv.70028 PMC11498999

[19] K. Vette , C. Bareja , R. Clark , and A. Lal , “Establishing Thresholds and Parameters for Pandemic Influenza Severity Assessment, Australia,” Bulletin of the World Health Organization 96, no. 8 (2018): 558–567. 30104796 10.2471/BLT.18.211508 PMC6083389

[20] A. Rguig , I. Cherkaoui , M. McCarron , et al., “Establishing Seasonal and Alert Influenza Thresholds in Morocco,” BMC Public Health 20, no. 1 (2020): 1029. 32600376 10.1186/s12889-020-09145-y PMC7323370

[21] R. Pung and V. J. M. Lee , “Implementing the World Health Organization Pandemic Influenza Severity Assessment Framework—Singapore's Experience,” Influenza and Other Respiratory Viruses 14, no. 1 (2020): 3–10. 31622034 10.1111/irv.12680 PMC6928060

[22] S. Muhemedi , P. Lusamba , J. C. Changachanga , L. Lubula , E. Nkwembe , and P. Babakazo , “The Application of Indicators to Assess the Severity of Seasonal Influenza Epidemics in Democratic Republic of Congo, 2015 to 2019,” Open Journal of Respiratory Diseases 12, no. 1 (2022): 1–14.

